# Utilization of Grape Seed Extract as a Natural Antioxidant in the Technology of Meat Products Inoculated with a Probiotic Strain of LAB

**DOI:** 10.3390/foods9010103

**Published:** 2020-01-19

**Authors:** Justyna Libera, Agnieszka Latoch, Karolina Maria Wójciak

**Affiliations:** Department of Animal Raw Materials Technology, Faculty of Food Science and Biotechnology, University of Life Sciences in Lublin, Skromna Street 8, 20-704 Lublin, Poland; justyna.libera@up.lublin.pl (J.L.); karolina.wojciak@up.lublin.pl (K.M.W.)

**Keywords:** grape seeds, waste, utilization, antioxidant, dry-cured meat, oxidative stability, probiotic

## Abstract

Grape seeds have been evaluated for use as food ingredients with stabilizing effects in meat technology. A pork neck, inoculated with probiotic monoculture (*Lactobacillus rhamnosus* LOCK900), was used as the matrix. The study compared the antioxidant potential of grape seed extract to sodium ascorbate. Three experimental variants of the products were prepared: With grape seed extract, with sodium ascorbate, and without additives. The meat ripened for two months, and during this period of time biophysicochemical analyses (product color, pH, number of lactic acid bacteria, content of free fatty acids, and thiobarbituric acid reactive substances) were carried out. It was found that the extract inhibited lipid hydrolysis occurring in the neck (1% of oleic acid) and limited oxidative processes (0.46 mg MDA kg^−1^), with efficacy similar to that of sodium ascorbate (0.9% of oleic acid and 0.53 mg MDA kg^−1^, respectively). No limitation of the desired lactic acid bacteria growth (approximately 7 log cfu g^−1^) was noticed in the meat samples with the extract. The results are optimistic because they indicate that not only is it possible to produce fermented pork neck inoculated with probiotic, but there are also no obstacles to utilizing grape seed extract as a natural antioxidant in this technology.

## 1. Introduction

Grape seeds, which are by-products of juices and wine production, are a rich source of biologically active compounds [1,2,3,4]. The seeds account for about 5% of the weight of the whole grape, representing approximately 40–50% of solid wastes that different wine industries generate during winemaking process [5]. The polyphenol compounds present in grape seeds can be used as a source of biologically active compounds, mainly antioxidants, in the technology of innovative meat products: Sausages [6,7,8,9] and other meats [10,11,12,13,14]. Wine production residues are rich in phenolic compounds [2,4,15,16], and seeds could be a valuable source of antioxidant substances, so it seems necessary to try to reuse this waste. According to Shi and co-workers [17], these compounds are able to trap and quench free radicals, and their antioxidant potentials have been shown to be four to five times higher than that of vitamin C or E. Tang and co-workers [1] noticed that the total flavonoid content in grape seeds is up to ten times higher than grape peel, thus reinforcing the importance of this grape by-product.

The main grape seed phenolic compounds include anthocyanins, flavan-3-ols, flavonols, stilbenes, and phenolic acids [4]. The total phenolic compounds content varies from 55 to 964 mg per 100 g of grape seeds, the average being 380 mg per 100 g of dry mass [18]. The average proanthocyanidins content, which is the most important component of grape seeds, is 159 mgg^−1^ of seed [19]. Grape seeds are proanthocyanidins, mainly composed of monomeric catechin and epicatechin, gallic acid, and polymeric and oligomeric proanthocyanidins [8,16], which have been demonstrated to be more powerful free radical scavengers than vitamins C, E, and β-carotene [20].

Grape seeds are an interesting alternative to conventional antioxidants in food technology, which is why the meat industry is also beginning to concentrate on the identification and bioactivity evaluation of grape by-products. The results of previously published studies indicate that grape seed extract (GSE) reduced oxidation in both raw and cooked meat, and that when added to pork it did not show significant antimicrobial activity of GSE relative to control [21]. This is an important observation, because it indicates the possibility of using GSE in the technology of fermented meat products with the use of probiotic bacteria. Other studies also indicate that phenolic compounds inhibit the growth of pathogenic bacteria, while they stimulate the growth of probiotics [22,23].

Potentially probiotic dry-fermented neck is a meat product made of a whole muscle running from the neck to the fifth thoracic vertebra of pork, and consists mainly of the muscles of the neck and part of the *Longissimus dorsi* muscle. The product is manufactured by curing, fermenting, drying, and ripening for at least several months. The neck muscles are a cut out of the pork carcass with a high fat content, which directly affects the palatability of meat products made from them. The quality and stability of dry-fermented neck depends on the course of physicochemical changes during the long time of ripening. As a result of the lipid oxidation, an undesirable, rancid odor and taste, as well as valuable polyunsaturated fatty acids occur in meat and vitamins are lost [16,24]. The microorganisms present in raw meat and starter culture affect the final quality too [25]. To limit the adverse oxidative changes in the dry-fermented meat, it is possible to use the special starter bacteria in the fermentation of meat and substances with antioxidant properties. Among the naturally occurring antioxidants in plants, there are rosemary, sage, thyme, aloe, mustard, etc. [11,14,26].

*Lactobacillus rhamnosus* LOCK900 strain belongs to a probiotic starter culture, which is used for the fermentation of meat products [27,28,29]. This monoculture has perfect probiotic properties; it exhibits excellent gastric acid and bile tolerance and has a high stability in foods [30]. However, some research results show that the metabolites produced by these bacteria can accelerate the oxidative changes that occur in the meat product during ripening, ageing, and storage [28,29].

Due to the fact that there are no scientific publications on the problem of using grape seed extract in the process of fermenting pork with probiotic, this study was undertaken to determine the oxidative stability and survival of bacteria in neck inoculated with *Lactobacillus rhamnosus* LOCK900 probiotic strain.

## 2. Materials and Methods

### 2.1. Grape Seed Extract Preparation

The extract was prepared by the authors, from seeds (post-production waste) obtained from a local wine producer from south-eastern part of Poland. The seeds were washed under a stream of water and dried at 40 ± 5 °C for 6 h. Then, 30 g of seeds, ground in grinder (Ronic Partner, Łódź, Poland), and 200 mL of 40% (*v*/*v*) ethanol were shaken for 2 h by a laboratory shaker (Water Bath Shaker Type 357, Elpan, Lubawa, Poland) at 150 rpm. The water bath temperature was 50 ± 10 °C. The slurry was thoroughly separated in an MPW-350R centrifuge (MPW Med. Instruments, Warsaw, Poland) at 5000 g for 15 min. The supernatant, which remained about 130 mL, was transferred to a round-bottom flask and concentrated in a vacuum evaporator (Rotavapor R-215, Buchi, Flawil, Switzerland) in a water bath at 50–60 °C and 100–175 hPa pressure. After evaporating 90% of the solvent, the genuine extract was formed in a final volume of about 13 mL. The extract was then sealed tightly in conical flask placed in the freezer. The grape seed extract was thawed immediately before use.

**Figure 1:**
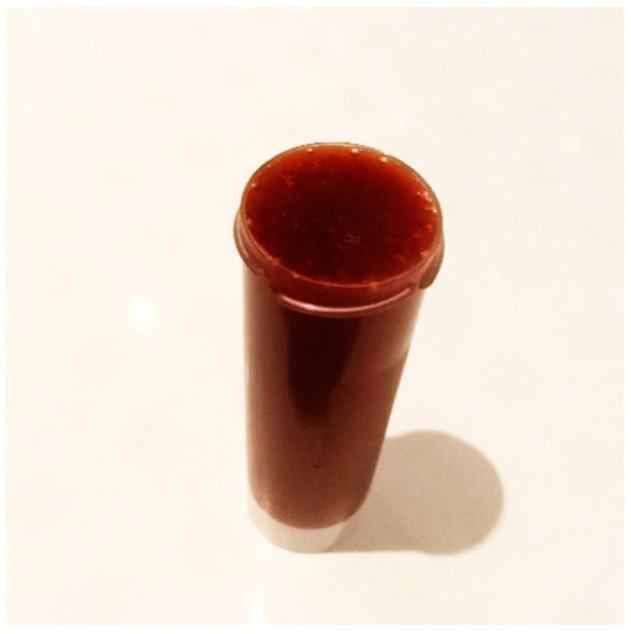
Grape seed extract prepared by authors.

As a result of this process, 1 g of extract was obtained from 2.3 g of raw seeds, so the drug extract ratio (DER genuine) was 2:3. Because the genuine extract had a compact consistency, it was added to the meat in a diluted form (Figure 1). Each extract portion (2 g kg^−1^ meat) was diluted immediately before use with distilled water to a volume of 4 mL. This concentration of GSE was selected as effective, based on our own research results that concerned spontaneously fermented meat without the addition of probiotics [31].

#### 2.1.1. Color Evaluation of Grape Seed Extract

The color of an extract was examined using the instrumental method—reflectance (X-Rite Color 8200 spectrophotometer, X-Rite Inc., Grand Rapids, MI, USA). The conditions were 13 mm port size, illuminant D65 and 10° standard observer. The X-Rite’s white and black standards were used to calibrate the spectrophotometer. To determine the color of the extract, it was previously poured into a clean beaker made of transparent glass, and then a beaker was placed into the measuring gap. Color results were determined in the CIEL*a*b* scale and lightness (L*), redness (a*), and yellowness (b*) were calculated [32].

#### 2.1.2. Total Phenolics Contents (TPC) of Grape Seed Extract

The amount of total phenolics was determined using Folin–Ciocalteau reagent [33]. To 0.5 mL of the sample, 0.5 mL of water, 2 mL Folin–Ciocalteau reagent (1:5 H_2_O) were added, and after 3 min, 10 mL of 10% Na_2_CO_3_. The contents were mixed and allowed to stand for 30 min. The absorbance at 725 nm was measured in a UV–Vis spectrophotometer (U-5100 UV-Vis, Hitachi High Technologies America Inc., Schaumburg, IL, USA). A calibration curve was generated using gallic acid as standard. TPC yield was expressed as a milligram of equivalent gallic acid (GAE) per milliliter of extract (mg GAE mL^−1^).

#### 2.1.3. Trolox Equivalent Antioxidant Capacity (TEAC) Assay of Grape Seed Extract

For measuring the antioxidant potentials of compounds present in the plant extract, the Trolox Equivalent Antioxidant Capacity (TEAC) assay was used [34]. A high TEAC value indicates that the mechanism of antioxidant action of extracts was as a hydrogen donor, and it could terminate the oxidation process by converting free radicals to the stable forms. ABTS radical cation (ABTS^+^) was produced by reacting 7 mmol ABTS solution with 2.45 mmol potassium persulphate and allowing the mixture to stand in the dark at room temperature for 12–16 h before use. The ABTS*^+^ solution was diluted with ethanol to an absorbance of 0.70 ± 0.02 at 732 nm. After the addition of 3.9 mL of diluted ABTS*^+^ solution to 100 µL of sample or Trolox standard, the absorbance was measured at exactly 6 min.

### 2.2. Probiotic Monoculture Preparation

Pure cultures of *Lb. rhamnosus* LOCK900 were obtained from the Pure Culture of the Technical University, Łódź, Poland (strain deposit number: CP005454). The inoculum of bacteria was prepared at the Chair of Food Hygiene and Quality Management (Warsaw University of Life Sciences, Warsaw, Poland) according to the procedure previously described by Neffe-Skocińska et al. [27]. The probiotic bacteria count in one milliliter of meat broth at the time of addition to meat was approximately 9.0 log cfu. For the meat fermentation, 2 mL of probiotic monoculture inoculums per kilogram of meat were used, which was the equivalent of 9.3 log cfu.

### 2.3. Manufacture of Dry-Fermented Necks

The production of dry-fermented pork necks consisted of four stages: Curing and antioxidant adding (stage 1), probiotic inoculation (stage 2), drying and fermentation at 16 °C for 21 days (stage 3), and ripening in vacuum packaging at 4 °C for 2 months (stage 4). The product was tested three times: After completing stage 3 (before ripening), during stage 4 (after the 1st month of ripening), and after completing stage 4 (after 2nd month of ripening). Two independent experimental trials were conducted. All determinations were performed in quadruplicate.

The study was carried out on pork meat cuts of Polish White Large breed. Necks (*M. longissimus cervicis*) were excised at 24 h post-mortem from half-carcasses chilled at 4 °C. At 72 h post-mortem, each piece of neck was divided into 3 equal parts (weighing about 1 kg each) and the meat underwent curing using a surface massage with a curing mixture composed of curing salt (56%), sea salt (43%), and sodium nitrate (1%). Antioxidant additives were applied together with curing mixture, and then three experimental variants were obtained: GSE—with grape seed extract (at the amount of 2 g kg^−1^of meat), ASC—with sodium ascorbate (the amount of 1 g kg^−1^ of meat), and CON—without antioxidant added (Table 1).

**Figure 2:**
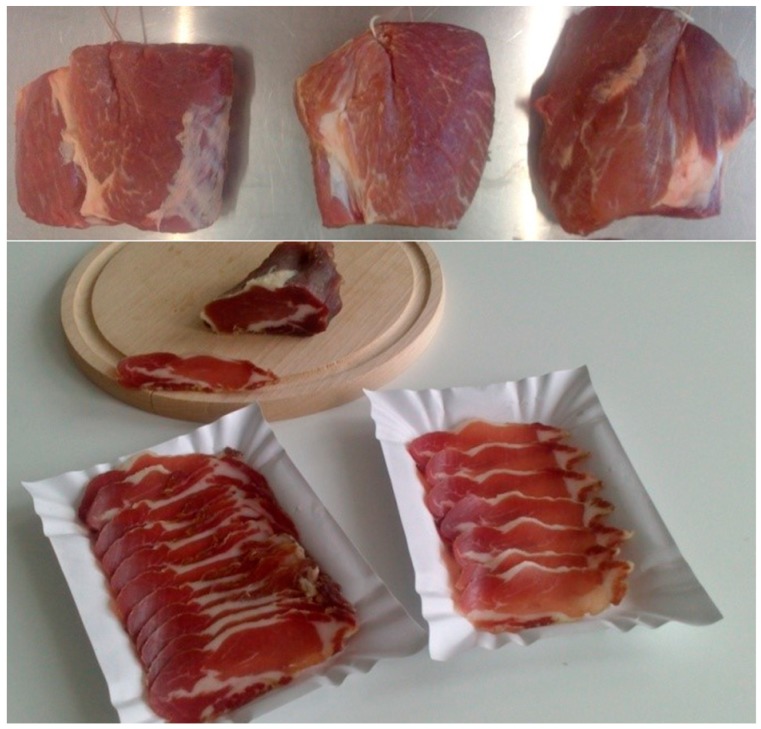
Dry-fermented pork neck: Before fermentation and ripening (**top**) and after two months of ripening (**bottom**).

Specified amounts of grape seed extract and sodium ascorbate were diluted in 4 mL of distilled water and additives were applied by rubbing them into the meat surface for about 3 min. Subsequently, all batches were placed in a refrigerator at 0 ± 1 °C for 72 h to allow the additives to diffuse. After curing, each piece was rubbed with glucose in the form of powder. Next, the probiotic starter monoculture (*Lb. rhamnosus* LOCK900) was applied; for this purpose, an automatic pipette equipped with sterile disposable tips was used. A measured quantity of inoculums—2 mL (9.3 log cfu) kg^−1^—was rubbed by hand into the meat. After that, the neck portions were hung (Figure 2, top) in a fermentation chamber under controlled conditions for 21 days. The conditions were: Temperature of 16 ± 1 °C, relative humidity 75 ± 5%, and air flow rate 0.05 m s^−1^ (30% of air circulation). Following fermentation and drying, the necks (weighing about 0.7 kg each) were individually vacuum-packed into polyethylene barrier bags and after that they ripened in a storage chamber at 4 ± 1 °C for 2 months. Figure 2 (below) shows the cross-sectional appearance of ready dry-fermented pork neck after ripening for 2 months.

### 2.4. Microbiological Characteristics of Dry-Fermented Neck

#### 2.4.1. Lactic Acids Bacteria (LAB) and Enterobacteriaceae (ENT) Counts of Meat Product

The measurements of the number of lactic acid bacteria—including probiotic bacteria and *Enterobacteriaceae*—were performed, and the counts were expressed as colony forming units (cfu) in grams. Samples for the microbiological analyses have been taken as a cross-section from the middle of the neck being tested. Microbiological analyses were carried out at AGROLAB Laboratory (Dęblin, Poland) according to ISO 15214 [35] and ISO 21528-2 [36].

#### 2.4.2. pH Value of Dry-Fermented Meat Product

The pH value was measured in a filtrate by mixing 10 g of minced sample with 100 mL of distilled water for 1 min, using a dispenser (T25 Basic Ultra-Turrax, IKA, Staufen, Germany). The pH was measured with a digital pH-meter CPC-501 (Elmetron, Zabrze, Poland) equipped with a pH electrode (ERH-111, Hydromet, Gliwice, Poland). The pH readings were recorded at exactly 4 min after the insertion of the electrode into the sample. The pH-meter was standardized with buffer solutions at pH 4.0, 7.0, and 9.0 [37].

### 2.5. Assessment of Oxidative Stability of Dry-Fermented Neck

#### 2.5.1. Free Fatty Acids (FFA) of Meat Product

The free fatty acids (FFA) of the meat product were determined using a previously described method [38] with some modifications [39]. Exactly 10 g of ground meat sample was blended for 2 min with 60 mL of chloroform in the presence of about 10 g of anhydrous sodium sulphate. Next, it was filtered through Whatman no.1 filter paper. Twenty mL of chloroform extract was dried in an SPU-200 oven (ZUT Colector, Cracow, Poland) to determine the fat weight. Another 20 mL extract was taken into a 150 mL conical flask. About 4 drops of 0.2% phenolphthalein indicator was added to the chloroform extract, which was titrated against potassium hydroxide solution (0.1 mol L^−1^ in 96% ethanol) to get the pink color end point. The quantity of potassium hydroxide consumed during the titration was recorded. FFA as percentage of oleic acid was calculated as follows:

$$\mathrm{FFA}\;(\%\;\mathrm{of}\;\mathrm{oleic}\;\mathrm{acid})\;=\;\frac{0.1\;\cdot \;0.1\;\mathrm{mol}\;\mathrm{KOH}\;\left(\mathrm{mL}\right)\;\cdot \;0.282\;}{\mathrm{weight}\;\mathrm{of}\;\mathrm{fat}\;\left(g\right)}\;\cdot \;100$$

#### 2.5.2. Thiobarbituric Acid Reactive Substances (TBArs) of Meat Product

TBArs were determined as in the method described by Pikul et al. [40], with slight modifications. Exactly 4 g of ground meat sample was blended for 1 min with 12 mL of cold perchloric acid (4%) in the presence of 200 μL of butylated hydroxytoluene solution (0.01% in ethanol). Then, it was filtered through Whatman no.1 filter paper. Subsequently, 650 μL of filtrate and 2-thiobarbituric acid solution (0.02 mol L^−1^ in distilled water) were mixed. A mixture of 650 μL of 4% cold perchloric acid and 650 μL of TBA solution was used as the reference sample. The heating part of the experiment was performed for 20 min at 100 °C. Then, spectrophotometrically (U-5100 UV-Vis, Hitachi High Technologies America Inc., Schaumburg, IL, USA) the absorbance at 532 nm was measured. The TBArs value as mg malondialdehyde per kg was calculated as follows:

TBArs (mg MDA kg^−1^) = 5.5 ∙ Absorbance at 532 nm

### 2.6. Color Evaluation of Meat Product

The color of dry-fermented neck was measured using an X-Rite Color 8200 spectrophotometer (X-Rite Inc., Grand Rapids, MI, USA) [32]. Color measurements were performed at room temperature at six different locations of neck slices. For the meat product, the measurements were made on a freshly cut surface of 10 mm thick slices. The total color difference (ΔE*) was calculated based on ΔL*, Δa* and Δb* results for each sample according to the time of ripening, as follows:

ΔE* = ((ΔL*)^2^ + (Δa*)^2^ + (Δb*)^2^)^1/2^

### 2.7. Statistical Analysis

For the statistical analysis, the collected data were analyzed by a two-way analysis of variance (ANOVA). The effect of added antioxidant and storage time on changes in selected physicochemical parameters was taken into account. The experiment was realized in four repeats in each of the replications. Calculations were made using Microsoft Office Excel 2007 (Microsoft Corporation, Redmond, WA, USA) and Statistica 10 (StatSoft, Warsaw, Poland) software. The normality of distribution of the variables within groups was verified with the Shapiro–Wilk test, and the Levene’s test was used to assess the equality of variances for a variable calculated for the groups. All the groups show the normality of distribution of the variables at *p* > 0.05. Next, the post hoc Tukey’s procedure was used to find patterns and relationships between subgroups. Differences among groups were determined as statistically significant at a level of *p* ≤ 0.05. All the results are expressed as means ± standard deviation.

## 3. Results and Discussion

### 3.1. Grape Seed Extract Characterization

An extract obtained by the authors was subjected to screening for its stability of color. The total color difference (ΔE*) was calculated from the difference of parameters before and after the sunlight exposure. The stability of color was determined for each sample according to the equation:

ΔE* = ((ΔL*)^2^ + (Δa*)^2^ +(Δb*)^2^)^1/2^

where: ΔL*, Δa* and Δb* are changes between values over time. The obtained values (ΔL* = 0.6; Δa* = 0.02 and Δb* = 0.1) indicate a very permanent color of the extract when in contact with sunlight (Table 2). It was ascertained that the colour of the applied extract was yellow-orange and looked just like buckwheat honey.

For measuring the potential antioxidant properties of the extract, the TPC and TEAC assays were used. It is known that the antioxidant capacity of food is strongly correlated with free phenolic content. The test showed that the extract prepared by the authors, possessed free radical-scavenging activity (Table 2). The TPC values obtained for the statement differ from the results of other authors. The results of papers by other authors showed that there could be about 100 times more total polyphenols compound in a commercial, lyophilized preparation from grape seed [21]. This discrepancy is probably due to the way we obtained the extract. The extract prepared by the authors was concentrated in a vacuum evaporator and then not freeze-dried, because the goal was to prepare the extract as simply as possible. Grape seed extract prepared by concentrating (but without freeze-drying) can be more likely used in meat technology because meat factories are not equipped with lyophilizers as standard.

### 3.2. pH Value, LAB, and Enterobacteriaceae Counts of Dry-Fermented Neck

The use of grape seeds in the meat technology with the addition of probiotic bacteria is not easy. A strategy should be planned in the production process that would allow the probiotics to grow in the meat matrix. The reformulation of the recipe, consisting of replacing sodium ascorbate with a plant extract, can lead to the inhibition of the development of beneficial microorganisms, which in turn can lead to beneficial, but also undesirable changes in the final product.

Grape seed extract has antioxidant and antimicrobial activity [22,23,41,42]. Antibacterial effects of the flavonoids and phenolics extracted from grape seeds are reported in literature [42,43,44]. The results showed that grape extract can limit the development of several pathogens, including: *Staphylococcus aureus*, *Bacillus subtilis*, *Psudomonas aeruginosa*, *Escherichia coli*, *Enterococcus faecalis Streptococcus pyogenes*, *Campylobacter* spp., etc.

The addition of plant material in meat technology can limit or eliminate the development of saprophytic or pathogenic bacteria, but it can also inhibit the development of probiotics in the product and change the direction of biophysicochemical changes. In the case of raw-fermented ripening meats—the technology of which uses a hurdle method—some uncontrolled changes in the direction of ripening may threaten product safety.

The number of lactic acid bacteria in the product, and thus its pH value, indicates the evolution of fermentation. In the experiment, no unfavorable effect of the extract applied on the neck pH was found (Table 3). In all variants (GSE, ASC, CON), values were similar (5.4–5.8) and did not differ significantly (*p* ≤ 0.05). During storage, the pH value of meat products was reduced by about 5% and this was a significant change (*p* ≤ 0.05). The increase in product acidity was due to the starter culture activity. Probiotic strain *Lb. rhamnosus* LOCK900 used for fermenting the pork neck, produces significant amounts of organic acids modifying the pH of the meat product. In the experiment, the LAB number was approximately 5.7 log cfu g^−1^ before ripening, and 6.7–7.2 log cfu g^−1^ after ripening, with no significant differences (*p* ≤ 0.05) between the test variants (GSE vs. ASC vs. CON). During ripening, a different LAB number was observed between the ASC sample (6.4 log cfu g^−1^) and GSE sample (6.9 log cfu g^−1^), but these differences were not significant (*p* > 0.05). The results indicate that the grape seed extract used in the technology did not work anti-bacterially against the lactobacilli. Interestingly, the authors found that this extract limited the growth of *Enterobacteriaceae*. Before ripening, the GSE neck was found to have a significantly lower (by two logarithmic) number of these bacteria than the other samples. In addition, during the ripening process, the number of *Enterobacteriaceae* in the GSE neck decreased even further (<1.00 log cfu g^−1^). During two months of ripening in stored chambers at 4 °C, the number of *Enterobacteriaceae* was reduced in all samples.

Wójciak et al. [28] found that after the completion of fermentation with probiotic *Lb. rhamnosus*, the pH of the meat product was similar to that recorded in our study (5.62). They recorded a significantly higher logarithm of LAB—almost two orders—in the pork neck fermented using the same probiotic. Significantly higher pH values were found by other researchers. Wang et al. [13] found that in the matured GSE bacon, the pH was 6.14 after three weeks of storage, and immediately after ripening, it was higher than 6.23. Interestingly, in the control sample without antioxidant, the pH was even higher, reaching 6.56 after maturation and 6.37 after storage. Much lower (about 4.5) pH values were noted in the Czech sausage *Polican* after the end of the 21-day fermentation [6]. High acidity referred to both samples with grape seed extract and control ones. Results of microbial analysis of the pork neck in this study were similar to the acidity results. All samples showed an increase in lactic acid bacteria during storage of the meat product under restricted air access.

### 3.3. Oxidative Stability of Dry-Fermented Neck

Bacterial starter cultures used in the meat fermentation process may exhibit different effects. Ruiz-Moyano et al. [25] have shown that some starter cultures can increase the oxidative stability of the ripening sausage, while others can reduce it. In the experiment described in this work, an *Lb. rhamnosus* LOCK900 probiotic culture was used for fermentation because it is a good starter due to its resistance to conditions in the digestive tract [30]; however, it may have a prooxidative effect in pork meat products during long-term ripening [27,28]. The oxidation process has progressed in all samples over time. There was a statistically significant (*p* ≤ 0.05) effect of the addition of grape seed extract on lipid hydrolysis inhibition (Table 4).

In the GSE sample, a lower concentration of free fatty acids (FFA) was found, which was similar to that obtained in the ASC sample. Immediately after production, the free fatty acid content in GSE and ASC samples was 24% lower and 36% lower after storage as compared to the CON sample. The results show the effective reducing properties of antioxidants found in this grape seed extract. The ripening pork neck produced with antioxidant additives (GSE and ASC) was characterized by increased oxidative stability during storage as compared to the CON one. Both antioxidants inhibited unfavorable fat metabolisms with similar efficacy. Immediately following the production, the samples GSE and ASC revealed the concentration of compounds reacting with 2-thiobarbituric acid to be lower by approximately 19% than those in the CON sample. During the two-month ripening in storage chamber, a 31–39% difference in the TBArs index was found between samples containing the antioxidant additive and the control sample. The TBArs value after the complete ripening of the potentially probiotic neck with the grape seed extract addition was lower than the ASC sample. These results suggest that grape seed extract can be used as an ascorbate substitute to delay the formation of secondary oxidation products. Wang et al. [13] noticed a similar (0.38 mg MDA kg^−1^) TBArs index in dry-fermented bacon with a grape seed extract addition. Very similar results have been described by Lorenzo et al. [7], who have used grape seed extract in chorizo production. The addition of the extract affected the reduction of the amount of secondary fat oxidation products. On the other hand, in the control sample, as in the present experiment, about 0.78 mg MDA kg^−1^ was recorded by those authors following the storage of the meat product. Other authors reported similar values of the TBArs of the control sample and in the samples with the addition of grape seed extract of different concentrations [9,12,21].

### 3.4. Color Evaluation of Dry-Fermented Neck

The addition of grape seed extract (GSE) had no negative effect on the color change of meat products during the first two study periods (Table 5). Studies carried out after a two-month ripening time showed differences between tests containing antioxidant additives. In the GSE and ASC samples, the lightness of the meat product was found to be about 11% higher than in the control sample after the completion of the production stages. This parameter was significantly modified by the ripening period and decreased over time. The lightness of ripening neck produced without the antioxidant (CON) was changed by about 24% during its time being kept in the storage chamber when compared to the values obtained previously.

The GSE sample was found to be redder than the others (10.3 vs. 9.2), which was a significant difference (*p* ≤ 0.05) (Table 5). Lowering the CIEL* value while increasing the CIEa* denotes a change in the color of the meat product to dark red or brown. In this sample, the change of this parameter along with the length of processing time was also noted. In the remaining samples (ASK and CON), no change in the CIEa* parameter was observed over time, while in the sample with the extract, an increase by 28% of this parameter was noted. These results indicate the antioxidant effect of the extract. It stabilizes the color of the product by inhibiting the conversion of red myoglobin to brown metmyoglobin. The applied supplements and ripening duration did not modify the CIEb* parameter (Table 5). The yellow color component of meat products ranged from 11.1 to 13.0.

Analysis of the total color change (ΔE*) of the meat product during ripening showed that the color of the control neck was the least stable (Table 6). The ΔE* of this variant during the first month of ripening was 9.5 units, indicating a large deviation of color from the standard that had the pork neck immediately after production. In the next month, the total color change was 3.6 units, which also indicates a significant deviation from the color of the product after the first month of ripening. In total, the color of control sample changed by as much as 12 units throughout the entire ripening process.

In the GSE sample, the total color change was the smallest, both after the first (4.7 units) and after the second (1.1 unit) month. These values indicate average color deviations. However, the total color change during the entire maturing process for the GSE sample was close to the value of the ASC sample (5.5 vs. 6.1 units). Other authors have also noticed a change in the color of matured meat product depending on time. Other authors [7] recorded the dependence observed in this experiment for higher values of the CIEa* parameter in the GSE additive sample. On the 50th day of the experiment, they noticed that the chorizo red color parameter with GSE addition was about three units higher as compared to the control sample. On the other hand, the yellow chromaticity and the color lightness (CIEL*) was higher by five units.

In our experiment, we compared the differences in the total color of the potentially probiotic neck, depending on the antioxidant supplement used. The sample with the extract had a different (3.2–4.1 units) total color than the other samples, before the ripening period. However, this test had the most durable color during processing. After the complete ripening process, the changes between the samples were as follows: Samples GSE and ASC were similar (2.7 units), samples CON and others were different by 4.6–5.8 units.

## 4. Conclusions

It was found that the dry-fermented pork necks inoculated with probiotic strain, which ripened for two months, were characterized by the appropriate biophysicochemical parameters. The use of each of the proposed antioxidant substances improved the oxidative stability of the neck, and the use of a plant-derived additive did not negatively affect the characteristics of the product and the direction of its changes during processing. The addition of grape seed extract effectively inhibits the lipid hydrolysis processes in the dry-fermented pork neck, reducing the free fatty acid content after two months of ripening in storage chamber at 4 °C. The grape seed extract, with efficacy similar to sodium ascorbate, counteracts unfavorable oxidative changes in the neck during processing stages. The results indicate that the addition of grape seed extract did not limit the development of the number of lactic acid bacteria in the meat product. Therefore, it is possible to grow probiotic bacteria in dry-fermented necks as a matrix. The utilization of grape seed extract also had a positive effect on inhibiting the number of *Enterobacteriaceae* in the product, and finally on eliminating them from the product. It was also found that the addition of grape seed extract to the dry-fermented pork neck had a positive effect on the color of the product. The color of the cross-section was redder compared to the other test variants, and the total color change of the product during storage was the smallest. The results of the experiment show that it is possible to create an innovative functional meat product using waste, which is part of the ‘no waste’ trend. The utilization of by-products and waste, and the use of them as a source of new raw materials in meat technology, allows the design of a new meat product with probiotic properties which is, thus, a functional food. The obtained research results are promising; moreover, they give hope for the use of other strains of probiotic bacteria for the fermentation of rarely used raw meat materials, i.e., pork neck.

## Figures and Tables

**Table 1:** Samples of potentially probiotic dry-fermented pork neck (additives per kg of meat).

Sample	Curing Salt (g)	Grape Seed Extract (g)	Sodium Ascorbate (g)	Glucose (g)	Probiotic Monoculture (mL)
GSE	30	2	-	12	2 (9.3 log cfu)
ASC	30	-	1	12	2 (9.3 log cfu)
CON	30	-	-	12	2 (9.3 log cfu)

Explanatory notes: GSE—neck with grape seed extract; ASC—neck with sodium ascorbate; CON—neck without antioxidant.

**Table 2:** Color and antioxidant properties of grape seed extract (mean ± standard deviation).

Tests	Grape Seed Extract
*Color parameters of extract:*	
CIEL*	27.7 ± 0.5
CIEa*	0.75 ± 0.3
CIEb*	11.2 ± 0.2
ΔE*_during exposure to light_	0.61
*Antioxidant properties of extract:*	
TEAC Assay (mmol TE of mL^−1^)	5.14 ± 0.8
TPC Assay (mg GAE mL^−1^)	6.74 ± 0.2

Explanatory notes: CIEL*—lightness; CIEa*—redness; CIEb*—yellowness; ΔE*—total color difference; TEAC—Trolox Equivalent Antioxidant Capacity; TPC—Total Phenolics Content.

**Table 3:** Lactic acid bacteria and *Enterobacteriaceae* counts (log cfu g^−1^), pH values of dry-fermented pork neck (mean ± standard deviation).

	Sample	Before Ripening	During Ripening	After Ripening
Lactic acid bacteria	GSE	5.71 ± 0.30 ^aA^	6.88 ± 0.28 ^aB^	6.93 ± 0.31 ^aB^
	ASC	5.69 ± 0.29 ^aA^	6.38 ± 0.28 ^aB^	6.67 ± 0.29 ^aB^
	CON	5.81 ± 0.31 ^aA^	6.52 ± 0.26 ^aB^	7.15 ± 0.27 ^aC^
*Enterobacteriaceae*	GSE	2.75 ± 0.30 ^bA^	<1.00 ± 0.00 ^bB^	<1.00 ± 0.00 ^bB^
	ASC	5.32 ± 0.29 ^aA^	3.64 ± 0.25 ^aB^	1.99 ± 0.29 ^aC^
	CON	4.89 ± 0.30 ^aA^	3.94 ± 0.24 ^aB^	2.23 ± 0.26 ^aC^
pH value	GSE	5.75 ± 0.06 ^aA^	5.50 ± 0.07 ^aB^	5.45 ± 0.05 ^aB^
	ASC	5.66 ± 0.09 ^aA^	5.42 ± 0.08 ^aB^	5.40 ± 0.06 ^aB^
	CON	5.73 ± 0.11 ^aA^	5.47 ± 0.05 ^aB^	5.41 ± 0.07 ^aB^

Explanatory notes: GSE—neck with grape seed extract; ASC—neck with sodium ascorbate; CON—neck without antioxidant; ^a,b^ within each column (sample), means followed by a common lowercase letter are not significantly different (*p* > 0.05); ^A–C^ within each row (ripening time), means followed by a common capital letter are not significantly different (*p* > 0.05).

**Table 4:** Free fatty acid (% of oleic acid), TBA-reactive substances (mg kg^−1^) of dry-fermented pork neck (mean ± standard deviation).

	Sample	Before Ripening	During Ripening	After Ripening
FFA	GSE	0.43 ± 0.03 ^bA^	0.68 ± 0.03 ^bB^	0.96 ± 0.05 ^bC^
	ASC	0.41 ± 0.03 ^bA^	0.65 ± 0.05 ^bB^	0.93 ± 0.05 ^bB^
	CON	0.55 ± 0.04 ^aA^	1.03 ± 0.04 ^aB^	1.49 ± 0.03 ^aC^
TBArs	GSE	0.31 ± 0.03 ^aA^	0.36 ± 0.04 ^bA^	0.46 ± 0.05 ^bB^
	ASC	0.29 ± 0.05 ^aA^	0.34 ± 0.05 ^bA^	0.53 ± 0.04 ^bB^
	CON	0.37 ± 0.04 ^aA^	0.57 ± 0.05 ^aB^	0.72 ± 0.05 ^aC^

Explanatory notes: GSE—neck with grape seed extract; ASC—neck with sodium ascorbate; CON—neck without antioxidant; ^a,b^ within each column (sample), means followed by a common lowercase letter are not significantly different (*p* > 0.05); ^A–C^ within each row (ripening time), means followed by a common capital letter are not significantly different (*p* > 0.05).

**Table 5:** Color parameters of dry-fermented pork neck (mean ± standard deviation).

Sample		Before Ripening	During Ripening	After Ripening
CIEL*	GSE	48.63 ± 1.55 ^bA^	44.42 ± 1.46 ^aB^	44.19 ± 1.69 ^aB^
	ASC	51.35 ± 2.40 ^aA^	45.00 ± 1.87 ^aB^	45.63 ± 1.71 ^aB^
	CON	52.57 ± 1.46 ^aA^	43.15 ± 2.06 ^aB^	40.01 ± 1.46 ^aB^
CIEa*	GSE	10.26 ± 0.50 ^aA^	12.33 ± 0.66 ^aB^	13.36 ± 0.68 ^aB^
	ASC	9.18 ± 0.57 ^bA^	10.28 ± 0.82 ^bA^	11.08 ± 0.68 ^bA^
	CON	9.18 ± 0.55 ^bA^	10.27 ± 0.85 ^bA^	11.73 ± 0.53 ^bA^
CIEb*	GSE	11.67 ± 1.08 ^aA^	12.11 ± 0.93 ^aA^	12.32 ± 0.92 ^aA^
	ASC	12.96 ± 0.88 ^aA^	12.78 ± 0.82 ^aA^	12.14 ± 0.98 ^aA^
	CON	11.48 ± 0.67 ^aA^	11.92 ± 0.60 ^aA^	11.13 ± 0.65 ^aA^

Explanatory notes: GSE—neck with grape seed extract; ASC—neck with sodium ascorbate; CON—neck without antioxidant; CIEL*—lightness; CIEa*—redness; CIEb*—yellowness; ^a,b^ within each column (sample), means followed by a common lowercase letter are not significantly different (*p* > 0.05); ^A,B^ within each row (ripening time), means followed by a common capital letter are not significantly different (*p* > 0.05).

**Table 6:** Total difference of color of dry-fermented pork neck.

	Before Ripening (Time 0)	During Ripening (Time 1)	After Ripening (Time 2)
ΔE*_GSE-ASC_	3.20	2.23	2.70
ΔE*_GSE-CON_	4.09	2.43	4.64
ΔE*_ASC-CON_	1.92	2.04	5.75
	**GSE**	**ASC**	**CON**
ΔE*_time 1–time 0_	4.71	6.45	9.49
ΔE*_time 2–time 0_	5.45	6.08	12.82
ΔE*_time 2–time 1_	1.08	1.20	3.55

Explanatory notes: GSE—neck with grape seed extract; ASC—neck with sodium ascorbate; CON—neck without antioxidant; ΔE*_samples_—total difference of color between the samples; ΔE*_time_—total difference of color over time periods.
